# Cold storage effects on immature calliphoridae: Survival limits and forensic consequences for PMI estimation

**DOI:** 10.1111/1556-4029.70356

**Published:** 2026-04-29

**Authors:** Larissa Thans Carneiro, Janyra Oliveira‐Costa, Márcia Souto Couri

**Affiliations:** ^1^ Department of Entomology Universidade Federal do Rio de Janeiro (UFRJ) Rio de Janeiro Brazil; ^2^ Forensic Entomology Laboratory Instituto Médico Legal Afrânio Peixoto – Polícia Civil do Estado do Rio de Janeiro Rio de Janeiro Brazil

**Keywords:** blowflies, forensic entomology, larval development, postmortem interval, refrigeration, temperature

## Abstract

This study investigated the effects of continuous refrigeration (4°C ± 2°C and 50% ± 5% RH) on the development and survival of immature stages (eggs, first, second, and third instar larvae, and pupae) of four forensically important blowfly species (*Chrysomya megacephala*, *Chrysomya putoria*, *Hemilucilia segmentaria*, and *Lucilia cuprina*) compared to a control group (27°C ± 2°C and 50% ± 5% RH). Groups of approximately 500 individuals at each stage were exposed to refrigeration and the control treatment, with periodic verifications to assess development and survival. Statistical analysis (two‐way ANOVA and Šidák posthoc test) revealed significant differences (*p* < .0001) in development time and survival between the groups. Eggs and first instar larvae did not survive longer than 36 h under refrigeration. Third instar larvae showed greater resistance, with *L. cuprina* surviving for up to 612 h, entering quiescence without molting. The results highlight the significant impact of refrigeration on the development of these warm‐climate species, warning against potential imprecisions in time of colonization (TOC) estimations and, consequently, in postmortem interval (PMI) if refrigeration effects are not considered. The research emphasizes the need for species‐specific data and caution when applying linear development models in forensic entomology, especially in cases involving refrigeration.

**Highlights**

- Cold storage halts blowfly development; L3 survive longer.
- Prolonged L3 survival masks actual insect colonization time.
- Cold tolerance varies by fly species and developmental stage.

## 1 INTRODUCTION

For at least 170 years, forensic entomology, when applied to death cases, has been essential for determining the postmortem interval (PMI) [1, 2]. The PMI refers to the time interval between the victim's death and the moment when the body is discovered [2, 3, 4]. In this context, fly larvae that develop on a decaying body can be collected and constitute evidence as significant as any other vestiges present at the crime scene [5].

However, for about 10 years now, many authors believe that, in fact, forensic entomology estimates the time of colonization (TOC) by insects on corpses, which helps determine the minimum postmortem interval (minPMI) [6, 7, 8, 9, 10, 11]. After death, a variety of insects is quickly attracted to the body due to specific odors, such as dipterans, beetles, and other carrion‐eating insects. However, in most cases, dipterans are reported as the first insects to find and begin colonizing a body. Oviposition or larviposition on the corpse marks the beginning of a “biological clock,” which forensic entomologists use to estimate the minPMI by analyzing the development of immature stages and the community of insects present [6, 8, 10]. Estimating the time since insect colonization provides a minPMI used to infer the time of death. The use of forensic entomology to estimate the TOC, rather than the minPMI directly, recognizes that insects colonize a body after death, and the time required for this colonization must be considered in the PMI estimation. Exceptions may occur, particularly in cases involving ante‐mortem infestation or neglect. Therefore, explicit recognition of these assumptions is essential when interpreting entomological evidence [10, 12, 13].

Currently, during initial criminal investigations, when a body is found, specialized forensic experts collect the larvae of colonizing insects directly at the crime scene and quickly transport them to the forensic entomology laboratory for analysis. However, there are situations in which the body, still containing saprophagous insects, can remain refrigerated for several days prior to the autopsy [14]. In addition, it may not be possible to immediately transport the insect larvae to the laboratory; in these cases, the specimens are stored in refrigerators or in coolers with ice until they reach the laboratory [15, 16].

Exposing insects to low temperatures has an impact on their development, but the degree of this interference is still not entirely clear, as there is little research into how each insect life stage (egg, larval instars, and pupa) is affected when kept in cold environments for several hours or days [14, 15]. This period of time under refrigeration has negative consequences that are often underestimated when inferring the TOC. The refrigeration temperature is rarely exact or strictly controlled, which results in errors and deviations in the calculation of the TOC, which are difficult to correct [17, 18, 19]. Therefore, criminal investigations are seriously compromised, as a reliable estimate of the time of death becomes unfeasible.

The four blow fly species central to this study—*Chrysomya megacephala* (Fabricius, 1794), *Chrysomya putoria* (Wiedemann, 1818), *Hemilucilia segmentaria* (Fabricius, 1805), and *Lucilia cuprina* (Fabricius, 1775)—are members of the Calliphoridae family (Diptera) and are widely recognized as forensically important taxa [1, 2, 20, 21, 22]. Unlike many cold‐adapted species typically studied in temperate regions, these species are predominantly found in warm, tropical, and subtropical climate regions [1, 14, 20, 21, 22, 23]. Specifically, *C. megacephala* is widespread across the Americas, Africa, and the Pacific, thriving particularly in urban environments in Brazil [2, 17, 20, 21]. *C. putoria* was introduced to Brazil in the 1970s, while *H. segmentaria* and *L. cuprina* are highly relevant indicators in the Neotropical Region [2, 17, 20, 21, 22].

Based on the above, this study investigated the development of immature stages of forensically important flies subjected to refrigeration conditions, analyzing how exposure to low temperatures influences the life cycle of these insects. The experiments aimed to simulate the refrigeration of insects used in forensic investigations before reaching the forensic entomology laboratory, as forensic evidence collection protocols recommend refrigeration of these specimens, and medicolegal protocols recommend refrigerating bodies when autopsies cannot be performed within a few hours after death. We sought to assess how development and survival are affected when the immatures of flies (at different stages of development: eggs, first, second, and third instar larvae, and pupae) are exposed to continuous refrigeration under controlled conditions in the laboratory. As will be better described in the methodology, the colonies used in this investigation were established from specimens collected in the city of Rio de Janeiro, Brazil, providing a geographically relevant foundation for studying cold tolerance in these warm‐adapted populations.

## 2 MATERIALS AND METHODS

### 2.1 Insect breeding and maintenance

A stock colony of the species *C. megacephala* (Fabricius, 1794), *C. putoria* (Wiedemann, 1818), *H. segmentaria* (Fabricius, 1805), and *L. cuprina* (Fabricius, 1775) (Diptera: Calliphoridae) was established in the laboratory. The colony was maintained and reared according to the methodology recommended by Queiroz & Azevedo [20]. Specimens were collected to establish the colony at the Instituto Médico Legal Afrânio Peixoto (IMLAP), located in the city of Rio de Janeiro, Brazil—according to the methodology proposed by Mello et al. [21]. As will be explained below, for the experiments, groups of approximately 500 individuals were selected. To make this possible, the dipteran colony consisted of at least 10 entomological cages of each species (measuring 40 × 40 × 40 cm). Each cage contained about 250 individuals (males and females in a sex ratio of 1:1) from several generations and approximately six cohorts. Both larval instar and age in hours were recorded throughout rearing. Only after individuals reached the intended instar—under controlled conditions of 27°C and 70% RH—were they transferred to refrigeration.

### 2.2 Exposure of immature insects to low temperatures continuously

**FIGURE 1:**
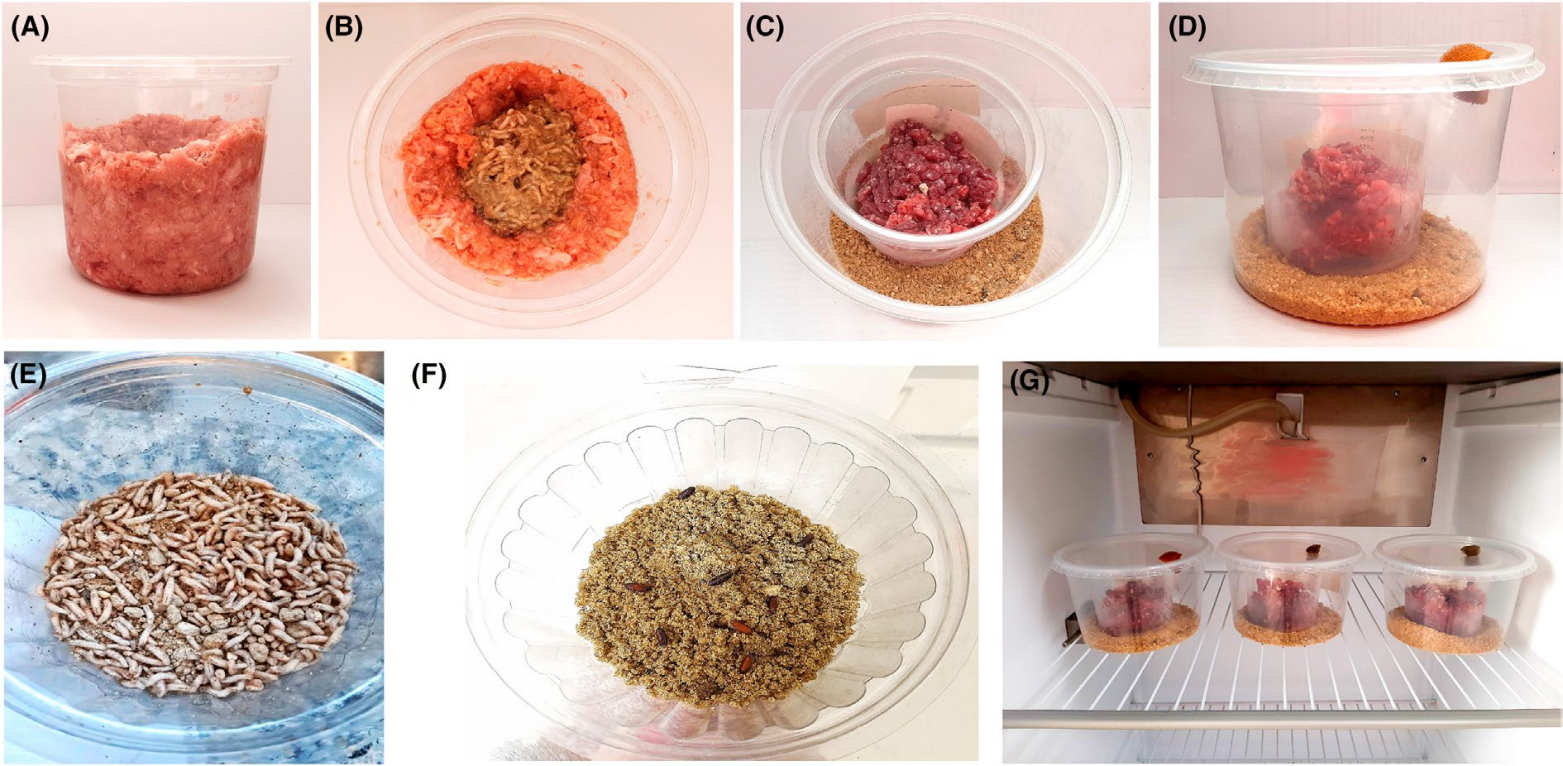
(A–D) Containers in which the immatures (eggs and larvae) were placed, along with the food substrate. (E, F) Containers in which errant larvae (3rd instar) and pupae were placed with sterilized sand. (G) Containers inside the climate chamber (BOD).

Groups of approximately 500 specimens, at different stages of development—eggs, first, second, and third instar larvae (named L1, L2. and L3, respectively, from independent cohorts)—were packed separately in polyethylene containers (700 mL) containing around 500 g of ground beef as a food substrate (1 g/larva ratio). These were inserted into larger jars (1 L) containing 2 cm of sterilized sand where the errant maggots would stay after abandoning the diet (Figure 1A–E). The pupae were placed in polyethylene containers (700 mL) containing only 2 cm of sterilized sand (Figure 1F). Subsequently, the immatures were kept in a climate chamber for bioassays (BOD—New Lab, model NL‐161‐01) under 4°C ± 2°C and relative humidity (RH) of 50% ± 5% until they had completed their development (Figure 1G). In total, six containers were used: three for the insects maintained under low temperatures continuously and three for the control group, which remained in the BOD (New Lab, model NL‐41‐02) at 27°C ± 2°C and a RH of 70% ± 5%. Both treatments were kept under a 12‐h photoperiod (photophase‐scotophase), starting at 6 a.m. In the control group experiment, the development of the insects was verified every 2 h; from the third larval instar onwards, the verifications were made every 12 h. In immatures kept under continuous refrigeration, verifications were made only every 12 h. During each verification, 30 specimens from each stage of development were removed from their containers from the larval masses, without any selection criteria, using entomological forceps and analyzed under a stereoscopic microscope (Feldmann Wild Leitz, model FWL—SMZ 7.5) to assess their development. The analysis was carried out as quickly as possible, and afterwards the analyzed larvae were returned to their containers in order not to impair their development.

Especially during the refrigeration treatment, to determine whether individuals would pupate or die, all specimens were monitored for more than 2 months, with repeated evaluations of activity and physical condition. Only after prolonged absence of movement and signs of tissue degradation (darkening, softening, necrosis) were individuals considered dead.

The effect of refrigeration on insect development was analyzed using a two‐way analysis of variance (ANOVA two‐way), since one way represented the experiments under refrigeration and the other the control treatments, obtaining a comparison between them; the Šidák correction was also carried out as a posthoc test to confirm significant differences. A 95% significance level was used for both tests, that is, “*p*” values <.05 were considered statistically significant. The Microsoft Excel program (version 2019) was used to analyze the raw data and the other analyses were carried out using GraphPad Prism software (version 10.0.2).

## 3 RESULTS

**FIGURE 2:**
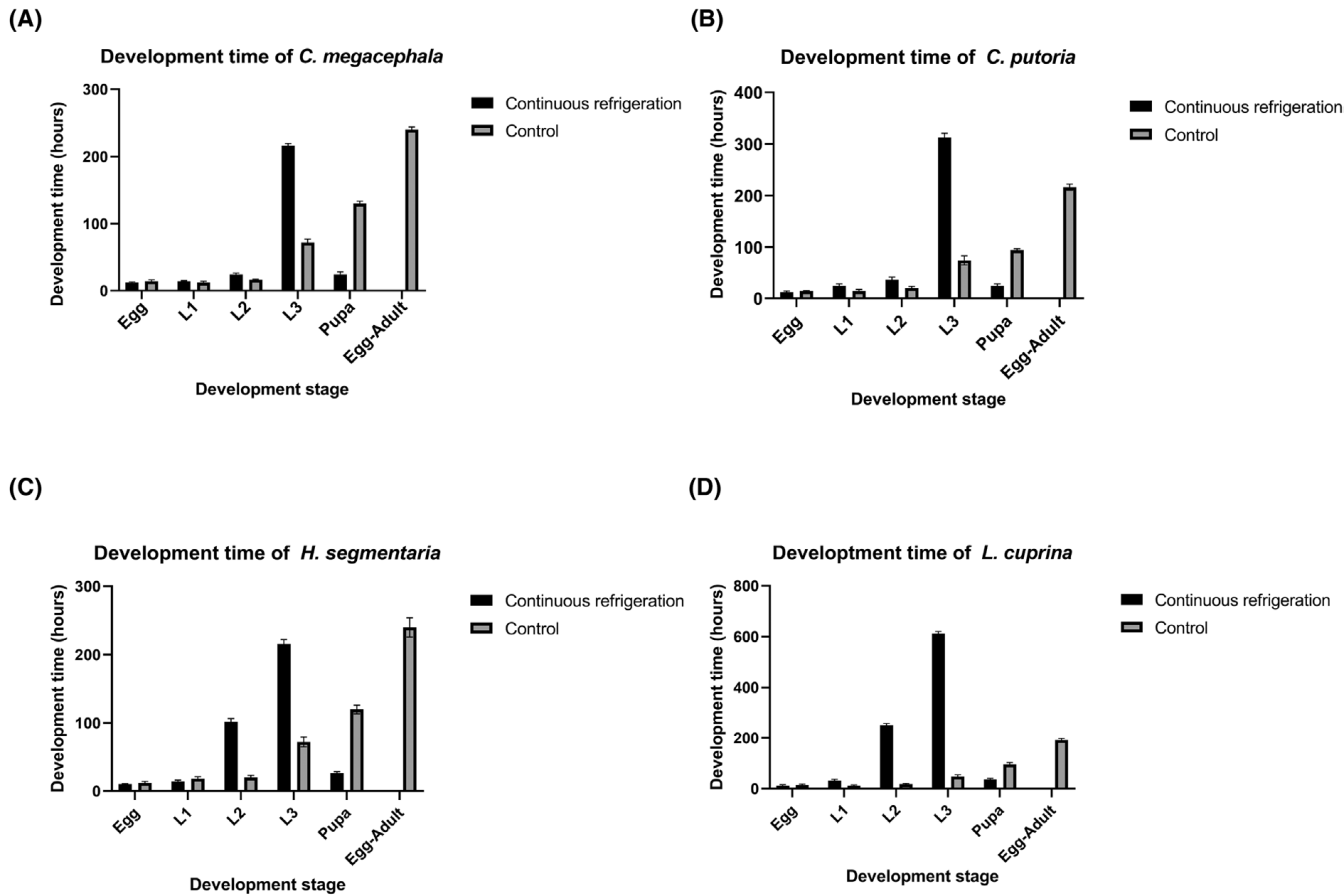
Comparison of average development time between treatments under continuous refrigeration and control. (A) Development time of *Chrysomya megacephala*. B: Development time of *Chrysomya putoria*. (C) Development time of *Hemilucilia segmentaria*. (D) Development time of *Lucilia cuprina*. In the graph, error bars represent standard deviation (SD).

When exposed to continuous refrigeration (4°C ± 2°C and RH of 50% ± 5%), after a period of approximately 12–14 h, the eggs of the species observed (*C. megacephala, C. putoria, H. segmentaria, and L. cuprina*) were already nonviable. In the control treatments (27°C ± 2°C and RH of 70% ± 5%), more than 90% of the eggs remained viable for around 12–16 h, with eclosion occurring after this period (Tables 1, 2, 3, 4 and Figure 2A–D). Under refrigeration, L1 larvae and pupae of all species also did not survive for more than 36 h (Tables 1, 2, 3, 4 and Figure 2A–D).

**TABLE 1:** Average development time (in hours ± standard deviation) of the immature stages of *Chrysomya megacephala* that were exposed to continuous refrigeration (4°C ± 2°C and 50% ± 5% RH) and the control treatment (27°C ± 2°C and 70% ± 5% RH). The *F*‐statistic, *p*‐values, and the percentage of total variation corresponding to the two‐way ANOVA analysis are also presented.

Development stage	Time (hours)		ANOVA two‐way	*F*‐valor	*p*	% of total variation
	Continuous refrigeration^a^	Control^b^				
Egg	12 ± 1.15^c^	14 ± 2.00	Interaction	479,082	<.0001	53.03
1st larval instar	14 ± 1.10^c^	12 ± 2.00	Development stage	387,110	<.0001	42.85
2nd larval instar	24 ± 2.30^c^	16 ± 1.20	Development time	180,225	<.0001	3.990
3rd larval instar	216 ± 3.46^c^	72 ± 5.29				
Pupa	24 ± 4.16^c^	130 ± 3.50				
Egg‐adult	ˍ	240 ± 4.20				

*Note*: The average development time of all instars differed significantly between the treatments under ^a^continuous refrigeration and the ^b^control group according to the two‐way ANOVA and Šidák posthoc test with 5% confidence. ^c^the immatures did not survive beyond the time period informed, there was no change of instar.

**FIGURE 3:**
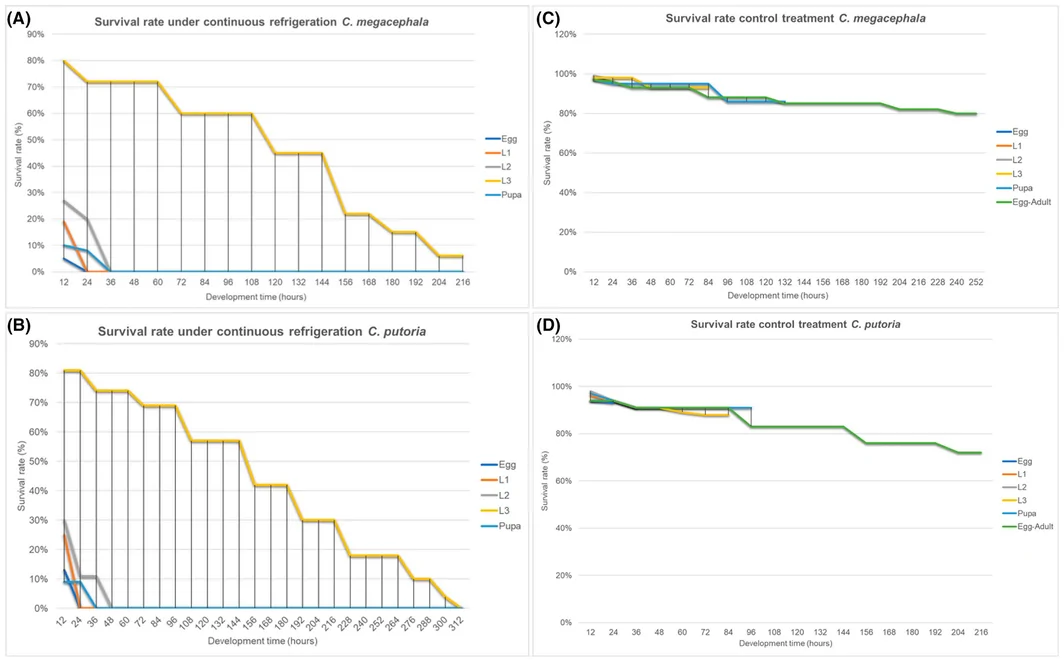
(A, B) Survival rate of *Chrysomya megacephala* and *Chrysomya putoria* under continuous refrigeration (4°C ± 2°C). (C, D) Survival rate of *Chrysomya megacephala* and *Chrysomya putoria* in the control treatment (27°C ± 2°C and RH of 70% ± 5%).

**FIGURE 4:**
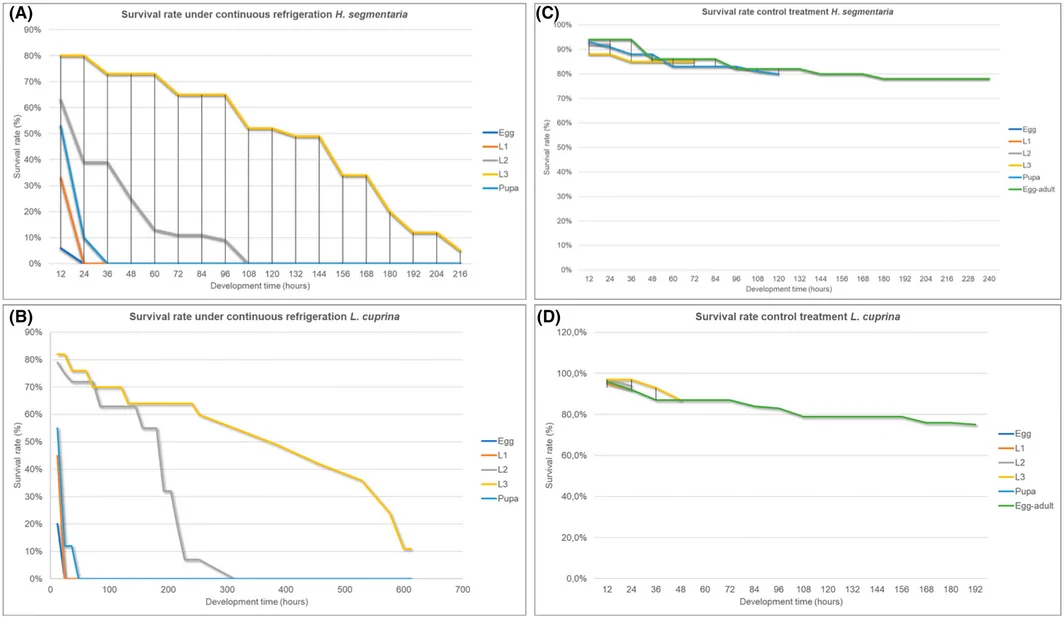
(A, B) Survival rate of *Hemilucilia segmentaria* and *Lucilia cuprina* under continuous refrigeration (4°C ± 2°C). (C, D) Survival rate of *Hemilucilia segmentaria* and *Lucilia cuprina* in the control treatment (27°C ± 2°C and RH of 70% ± 5%).

The two‐way ANOVA indicated significant differences (*p* < .0001) in the average development time and survival of the immatures exposed to continuous refrigeration in relation to the control treatments, with the greatest difference between the L2 larvae of *C*. *putoria* (Table 2, Figures 2B and 3B,D). *H. segmentaria* (Table 3; Figures 2C and 4A,C) and *L. cuprina* (Table 4; Figures 2D and 4B,D); and, above all, L3 larvae of all the species studied in this work, which was confirmed by the Šidák correction posthoc test.

**TABLE 2:** Average development time (in hours ± standard deviation) of the immature stages of *Chrysomya putoria* that were exposed to continuous refrigeration (4°C ± 2°C and 50% ± 5% RH) and the control treatment (27°C ± 2°C and 70% ± 5% RH). The *F*‐statistic, *p*‐values, and the percentage of total variation corresponding to the two‐way ANOVA analysis are also presented.

Development stage	Time (hours)		ANOVA two‐way	*F*‐valor	*p*	% of total variation
	Continuous refrigeration^a^	Control^b^				
Egg	12 ± 2.31^c^	14 ± 1.15	Interaction	225,753	<.0001	52.56
1st larval instar	24 ± 4.00^c^	14 ± 3.05	Development stage	202,375	<.0001	47.12
2nd larval instar	36 ± 5.03^c^	20 ± 3.10	Development time	999.1	<.0001	0.04652
3rd larval instar	312 ± 8.72^c^	74 ± 9.02				
Pupa	24 ± 4.16^c^	94 ± 3.06				
Egg‐adult	ˍ	216 ± 6.11				

*Note*: The average development time of all instars differed significantly between the treatments under ^a^continuous refrigeration and the ^b^control group according to the two‐way ANOVA and Šidák posthoc test with 5% confidence. ^c^the immatures did not survive beyond the time period informed, there was no change of instar.

**TABLE 3:** Average development time (in hours ± standard deviation) of the immature stages of *Hemilucilia segmentaria* that were exposed to continuous refrigeration (4°C ± 2°C and 50% ± 5% RH) and the control treatment (27°C ± 2°C and 70% ± 5% RH). The *F*‐statistic, *p*‐values, and the percentage of total variation corresponding to the two‐way ANOVA analysis are also presented.

Development stage	Time (hours)		ANOVA two‐way	*F*‐valor	*p*‐valor	% of total variation
	Continuous refrigeration^a^	Control^b^				
Egg	10 ± 1.15^c^	12 ± 2.00	Interaction	144,847	<.0001	60.16
1st larval instar	14 ± 2.00^c^	18 ± 3.06	Development stage	91,306	<.0001	37.92
2nd larval instar	102 ± 4.62^c^	20 ± 3.10	Development time	17,097	<.0001	1.420
3rd larval instar	216 ± 6.11^c^	72 ± 7.02				
Pupa	26 ± 2.31^c^	120 ± 6.43				
Egg‐adult	ˍ	240 ± 14.04				

*Note*: The average development time of all instars differed significantly between the treatments under ^a^continuous refrigeration and the ^b^control group according to the two‐way ANOVA and Šidák posthoc test with 5% confidence. ^c^the immatures did not survive beyond the time period informed, there was no change of instar.

**TABLE 4:** Average development time (in hours ± standard deviation) of the immature stages of *Lucilia cuprina* that were exposed to continuous refrigeration (4 ± 2°C and 50 ± 5% RH) and the control treatment (27 ± 2°C and 70 ± 5% RH). The *F*‐statistic, *p*‐values, and the percentage of total variation corresponding to the two‐way ANOVA analysis are also presented.

Development stage	Time (hours)		ANOVA two‐way	*F*‐valor	*p*	% of total variation
	Continuous refrigeration^a^	Control^b^				
Egg	12 ± 3.46^c^	14 ± 4.60	Interaction	629,570	<.0001	52.45
1st larval instar	32 ± 5.00^c^	12 ± 3.06	Development stage	476,945	<.0001	39.73
2nd larval instar	252 ± 6.11^c^	18 ± 3.10	Development time	463,483	<.0001	7.722
3rd larval instar	612 ± 8.33^c^	48 ± 7.21				
Pupa	36 ± 4.62^c^	96 ± 7.02				
Egg‐adult	ˍ	192 ± 6.10				

*Note*: The average development time of all instars differed significantly between the treatments under ^a^continuous refrigeration and the ^b^control group according to the two‐way ANOVA and Šidák posthoc test with 5% confidence. ^c^the immatures did not survive beyond the time period informed, there was no change of instar.

When kept at low temperature, none of the immatures “molted,” but the L3 larvae resisted for a considerable time: 6% of the individuals of *C. megacephala* and 5% of *H. segmentaria* survived for around 216 h (Figures 3A and 4A); 4% of *C. putoria* for 312 h (Figure 3B); 11% of *L. cuprina* for 612 h (Figure 4B). In contrast, in the control treatments, the immatures showed reduced development time and much higher survival rates compared to the experiments under continuous refrigeration, with around 76%–98% of the individuals still alive in each of the different instars (Figures 3C,D and 4C,D).

For all species, the Sidak posthoc test also indicated that the average development time of each of the stages exposed to refrigeration also differed significantly from the average time of the stages in the control treatments (i.e. L1 under refrigeration versus L1 in the control treatments; L2 under refrigeration versus L2 in the control treatments and so on). The values can be seen in Tables 1, 2, 3, 4.

## 4 DISCUSSION

To accurately estimate the minPMI, it is essential to obtain precise temperature measurements to which developing insects were exposed at the crime scene, as temperature is one of the most critical factors affecting the development of necrophagous insects [19, 22, 23]. The growth rates of species such as blowflies can be represented as an asymmetrical “S” shaped curve: development is almost zero at low temperatures, increases linearly at medium temperatures, and decreases at high temperatures, up to a lethal point [19, 22, 23]. However, it is common for bodies to be stored in refrigeration until the time of the autopsy before insect samples are collected, which can have a significant impact on PMI calculations. Refrigeration can affect the TOC, the development of fly larvae and, consequently, minPMI estimates [17, 24]. Simulations of a refrigeration period at 4°C, varying from 24 to 240 h, for example, have shown that the time of exposure to low temperatures influences the rate of development after cooling [24]. The handling of corpses, as well as climatic and entomological data, are just as important as the collection of trace evidence at the crime scene. Therefore, it is essential to consider the effects of refrigeration when estimating the minPMI based on entomological evidence.

Our results indicate that refrigeration significantly delays the development of immature insects and can even halt it, which is consistent with findings from previous studies [14, 15, 16, 17, 23, 25, 26]. However, the majority of studies on the effects of temperature on insect development focus on species adapted to cold climates. In contrast, our work evaluates the impact of refrigeration on species such as *C. megacephala, C. putoria, H. segmentaria*, and *L. cuprina*, which are more commonly found in warm climate regions. This is significant because the response of these species to refrigeration may differ from that of species adapted to cold, making our results particularly relevant for forensic investigations in tropical and subtropical regions. Even within the same species, different genetic populations may exhibit varying levels of cold tolerance [23]. When working with species from warm climate regions, one may potentially uncover genetic variations that influence the capacity to adapt to refrigeration episodes. This could have significant implications for the accuracy of PMI estimates, as the response to cold may vary depending on the geographic origin of the insect population.

In addition to the delay in development, the present study also examines the survival rate of refrigerated larvae. This information is crucial, as the mere fact that a larva survives refrigeration does not imply that its development has not been affected. By combining data on development and survival, we provide a more comprehensive understanding of the impact of refrigeration on insect populations. At low temperatures, none of the juvenile forms underwent molting; however, the L3 larvae of all species and L2 larvae of *L. cuprina* persisted for a considerable duration. Myskowiak & Doums and Magni et al. [15, 16] also analyzed the effects of refrigeration on insect development and its implications for the estimation of the PMI in forensic investigations. However, our study differs by focusing on different species, with a methodology adapted to the procedures carried out by Brazilian law enforcement agencies and the limited technological resources available. Additionally, we examined further variables, such as survival rate and the effect of refrigeration on different stages of larval development, thus offering new perspectives and contributions to the field of forensic entomology.

It was observed that eggs became unviable within a few hours of refrigeration exposure (between 12 and 14 h), likely due to rapid desiccation at low temperatures and low RH, as reported by Magni et al. (2016) [16]. In our study, eggs were classified as nonviable when, after approximately 10–12 h, they exhibited marked desiccation, darkening of the chorion, or collapse. Dead larvae showed dark coloration, soft body consistency, and early decomposition. Lee and Sømme [25, 27] also found that eggs, immature stages, and small‐sized insects generally supercool at lower temperatures compared to adults or larger individuals. This phenomenon is related not only to size but also to body weight and water volume in insects. L1 larvae of all species did not survive, possibly because, even when aggregated, they could not generate enough metabolic heat to warm themselves and stay alive [16, 17, 19]. This reinforces the importance of considering larval mass and thermoregulatory effects in forensic investigations. The larval mass plays a crucial role in maintaining temperature and larval development, especially in environments with unfavorable temperatures. Aggregated larvae generate metabolic heat, raising the internal temperature of the mass and creating a microclimate more favorable to development [16]. This thermoregulation phenomenon is particularly important for first instar larvae (L1), which have a lower capacity to generate heat individually. Aggregation allows larvae to overcome physiological limitations and maintain active metabolism, prolonging their survival in refrigerated conditions. Similarly, it is believed that pupae were also unable to maintain functional metabolism at low temperatures. Additionally, a recent study demonstrates that exposure to low temperatures causes physiological damage and neuromuscular dysfunction in insects [28].

The present research faced logistical challenges in replicating large larval masses, which may have limited the ability to observe the thermoregulatory effect at its maximum potency. Creating large larval masses requires a significant volume of food substrate and rigorous control of environmental conditions, such as humidity and ventilation, to avoid the accumulation of metabolic waste and the proliferation of pathogenic microorganisms. The absence of large larval masses in our experiments may have led to an underestimation of the thermoregulation potential of the studied species and, consequently, to an incomplete understanding of their response to refrigeration. Other problems faced were the lack of space, materials, and more climate chambers for bioassays to conduct the experiments. In real forensic cases, bodies are often found wrapped in removal bags or with larvae colonizing natural orifices, such as the mouth, nose, and ears. These situations create microclimates that can significantly influence the temperature and humidity around the larvae. Removal bags can isolate the body from the external environment, slowing cooling and allowing larvae to maintain higher temperatures for longer. Natural orifices offer protection against desiccation and can house dense larval masses, with high metabolic activity and thermoregulation. The absence of these microclimates in our experiments may have exacerbated the negative effects of refrigeration and limited the generalization of the results to real forensic scenarios.

It was observed that L2 larvae, and especially L3 larvae, remained in a state of quiescence until their energy reserves were depleted, resulting in their death. This phenomenon, also reported by Myskowiak & Doums [15] in studies with *Protophormia terraenovae* (Robineau‐Desvoidy, 1830), demonstrates that refrigeration does not prevent larval survival, but induces a state of temporary metabolic inactivity, extending the time window in which colonization can be estimated. The refrigeration time directly influences the consumption of the larvae's energy reserves, while the temperature affects the metabolic rate during quiescence. Thus, the prolonged survival of larger larvae can mask the actual colonization time, impacting the accuracy of the minPMI. Myskowiak & Doums [15] found that exposing different instars to 10 days of refrigeration can lead to imprecisions of more than 6 h in the minPMI estimation. The application of development models and corrective calculations, such as Monte Carlo simulation, as proposed by Hill et al. [13], can assist in adjusting the minPMI estimation, taking into account the effects of refrigeration on larval development and allowing for a more accurate interpretation of entomological data in forensic contexts.

L3 larvae exhibited greater resistance to refrigeration, with *L. cuprina* showing the highest survival capacity, remaining viable for up to 612 h. This higher tolerance may be explained by the greater amount of energy reserves accumulated at this larval stage [19], allowing them to survive longer under adverse conditions [17, 26]. However, it is important to note that, although L3 larvae survive for an extended period, their development is halted, and no molting occurs, reinforcing the hypothesis of a state of quiescence or cold‐induced diapause.

The induction of diapause or quiescence in larvae exposed to low temperatures, as observed in this study, has significant implications for the estimation of the minPMI. Linear methods for calculating the PMI, which assume a constant development rate in relation to temperature, can lead to inaccurate estimates when larval development is interrupted by cold episodes [23]. This interruption in development must be considered when estimating the PMI, as it can generate inaccuracies in predictions based on these models. Linear models for minPMI estimation often do not incorporate the complexity of insect physiological responses to temperature variations, such as entering diapause or quiescence, so accurate determination of the lower development threshold temperature (minimum threshold) for each species is very important. The lack of accurate data on these thresholds can lead to erroneous interpretations of the effects of cold episodes on larval development [23]. The comparison with control treatments maintained at 27°C ± 2°C clearly demonstrates the importance of temperature in the “normal” development of flies. The control groups showed faster development and significantly higher survival rates at all stages of development [19, 29]. The body cooling rate is also an important factor. Bodies without larval infestation cool at a rate of about 1°C–2°C per hour, while bodies with larval infestation cool at a lower rate, about .12°C/h, and it can take up to 100 h to reach the minimum temperature of the refrigerated environment [30]. The time it takes for larvae to reach the lower development threshold temperature after refrigeration must be considered in the minPMI calculation. Therefore, research that aims to identify the real minimum threshold of different species is fundamental to assess the real effects of cold episodes and improve the accuracy of minPMI estimations as Ames & Turner have already pointed out [23].

Additionally, this study reveals variations in refrigeration tolerance among different species. *L. cuprina* exhibited greater cold resistance in the L3 stage compared to *C. megacephala*, *C. putoria*, and *H. segmentaria*. These interspecific differences may be attributed to physiological and behavioral adaptations specific to each species [26, 28], such as the ability to generate and retain heat within larval aggregations [16, 17, 19]. These variations highlight the need for species‐specific studies in forensic entomology.

As demonstrated in this study and others [13, 15, 17, 19, 26, 31], refrigeration can lead to loss of precision in minPMI estimation. Fluctuating temperatures in refrigerated chambers, lack of strict control, absence of precise measurements, and variations in storage conditions make correcting minPMI estimates difficult. Therefore, it is essential to assess the temperature at which bodies are kept, the duration of storage in a refrigerator, the developmental stage, and the species involved [13, 15, 19].

The use of thermal summation models to calculate minPMI can be inaccurate when temperature is not constant [13, 29]. The use of accumulated degree‐day (ADD) methods may not be suitable for simulating development conditions in refrigerators, as these methods assume that development occurs at a constant rate at temperatures above the minimum threshold. However, under low‐temperature conditions, development may be halted or drastically reduced, leading to minPMI underestimations [10, 13]. The minimum threshold ranges from 15°C to 10°C for necrophagous dipteran species from tropical and neotropical regions [22], but there are not many studies that assess the development of these species at temperatures below 10°C or below 4°C. We believe that further research on the thermal requirements of these species, including low temperatures, needs to be conducted; perhaps, with more data available, the ADD method could be applied more reliably.

Considering variations in crime scene temperatures and refrigerated environments is also highly important. Studies have shown that ambient temperatures can vary significantly depending on location, sun exposure, and other environmental factors [19, 31]. Body temperature may also vary depending on body mass, decomposition stage, and environmental conditions. Therefore, it is essential to record local and body temperatures to improve minPMI estimation accuracy [19, 31, 32].

In summary, our study demonstrated that continuous refrigeration negatively affects the development of flies in the Calliphoridae family and that the L3 larval stage shows greater tolerance to refrigeration. Refrigeration induces a state of diapause/quiescence that halts larval development and eventually leads to death. The different responses of each species to refrigeration emphasize the need for species‐specific studies in forensic entomology. The results highlight the importance of considering the temperature and refrigeration history of samples in minPMI estimation. It is essential for forensic entomologists to record storage conditions and refrigeration duration to enable a more reliable minPMI estimate. Standardizing body and insect sample storage conditions and using precise, case‐relevant temperature data are crucial for improving forensic investigation accuracy. Additional studies are needed to investigate the effects of refrigeration at different insect developmental stages, including the combination of different temperatures and the impact of storage conditions on minPMI estimation.

A limitation of this study was the unavoidable difference in RH between the experimental groups because of technical issues encountered with the equipment and limited resources. While the control group was maintained at 70% RH, the refrigeration group was kept at 50% RH to prevent external inoculative freezing too, which would have compromised the evaluation of metabolic quiescence. Future studies should aim to isolate these factors to better understand their individual contributions to larval mortality in refrigerated remains.

Future studies should also focus on replicating the conditions found at crime scenes, including the use of corpse removal bags and the simulation of natural orifice colonization. The use of experimental models with different sizes of larval masses and continuous monitoring of temperature and humidity in the created microclimates will allow for a more accurate understanding of the impact of refrigeration on larval development in relevant forensic contexts. Furthermore, the analysis of the expression of genes related to thermoregulation and cold tolerance may provide insights into the molecular mechanisms involved in the adaptation of larvae to low‐temperature environments as also proposed by Ullah et al. (2024) [28].

## FUNDING INFORMATION

Financial support provided by the Programa de Pós‐Graduação em Zoologia do Museu Nacional/UFRJ and the Coordenação de Aperfeiçoamento de Pessoal de Nível Superior (CAPES).

## CONFLICT OF INTEREST STATEMENT

The authors declare no conflicts of interest.

## Data Availability

The data that support the findings of this study are openly available in Depósito de teses e dissertações at https://biblioteca.museunacional.ufrj.br/normas/deposito‐de‐teses‐e‐dissertacoes/.
